# Evolution of Early-Phase Anticancer Drug Investigations in China

**DOI:** 10.1001/jamaoncol.2022.3856

**Published:** 2022-09-08

**Authors:** Shuhang Wang, Yue Yu, Yale Jiang, Huishan Zhang, Peiwen Ma, Gang Liu, Ning Li

**Affiliations:** 1 Clinical Trial Center, National Cancer Center/National Clinical Research Center for Cancer/Cancer Hospital, Chinese Academy of Medical Sciences and Peking Union Medical College, Beijing, China; 2 Phase I Clinical Trial Ward, Fujian Medical University Cancer Hospital and Fujian Cancer Hospital, Fujian Province, China; 3 Key Laboratory of Molecular Epigenetics of the Ministry of Education, Northeast Normal University, Changchun, China

## Abstract

This study examines phase 1 anticancer drug clinical trials performed in China from 2017 to 2021.

In recent years, China has implemented a revised drug administration law and registration regulation. The purpose is to accelerate the development of new innovative drugs. Policies, such as the *“*60 working day silent approval of investigational new drug applications” and “Technical Guiding Principles for the Acceptance of the Overseas Clinical Trial Data of Drugs,” have had a positive association with timelines for trial startup and marketing of new innovative drugs in China. These policies have been associated with significantly increased anticancer drug trials and more diversified anticancer therapies that benefit patients.^1,2^ In this article, we present our analysis of phase 1 clinical trials performed in China from 2017 to 2021.

## Methods

Details of oncology phase 1 trials in solid tumors were obtained from INFORMA database (https://pharma.id.informa.com). One-hundred sixty-one trials were excluded from the 1526 identified trials based on the exclusion criteria (eMethods in the Supplement). The average annual growth rates (AAGR) of trials were calculated as Z = (X/Y)^1/3-1. The variables X and Y represent the trial numbers in 2021 and 2017, respectively.

## Results

A total of 996 drugs were tested in phase 1 trials in China. Most drugs (461 [46%]) were immuno-oncology drugs (Figure, A), among which cell therapy (200 [20%]) constituted the largest category (Figure, B). Nine trials conducted in China during the 4-year period included first-in-class drugs with novel targets (Table). For example, GNC-035, GNC-038, and GNC-039 were the first tetra-specific antibodies targeting immune antigens; CBP-1008 was the first bispecific ligand drug conjugate.

**Figure.**
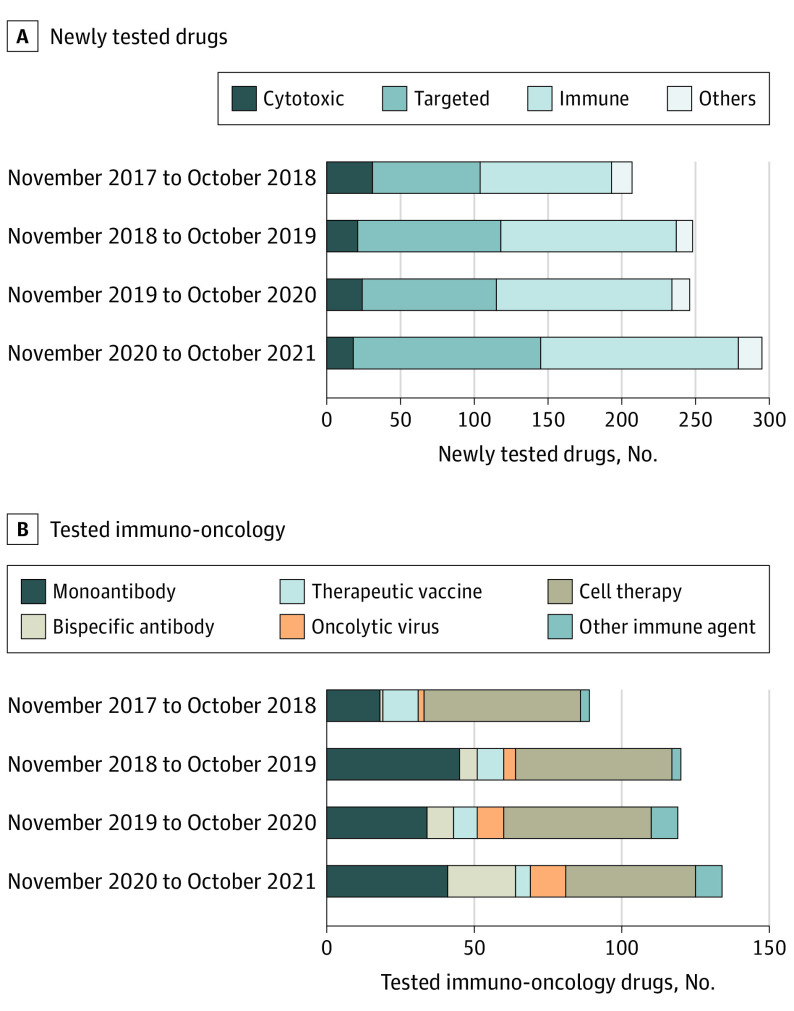
Anticancer Phase 1 Clinical Trials A, Types of tested drugs. B, Types of tested immune drugs.

**Table.** Novel Targets of Phase 1 Clinical Trials Performed in China

Novel target/target group	First-tested year		Type of drug	Specific drug	Sponsor (trials in China)
	Outside of China	In China			
ROR2	2018	2018	CAR-T cell	CCT301-59 CAR-T cells	Shanghai PerHum Therapeutics Co, Ltd
BTLA	2018	2020	Monoantibody	JS004	Shanghai Junshi Bioscience Co, Ltd
Nectin4/FAP dual target	2019	2019	CAR-T cell	Nectin4/FAP–targeted CAR-T cells	Sixth Affiliated Hospital of Wenzhou Medical University
TRPV6/FRα dual target	2019	2019	Bispecific ligand drug conjugate	CBP1008	Coherent Biopharma (Suzhou) Co, Ltd
TRAILR2/CDH17 dual target	2020	2020	Bispecific antibody	BI 905711	Boehringer Ingelheim
IRE-1α	2020	2020	IRE-1α inhibitor	ORIN1001	Fosun Orinove (Suzhou) PharmaTech
CD19/CD3/PD-L1/4-1BB	2020	2020	Tetra-specific Antibody	GNC-038	Sichuan Baili Pharmaceutical Co, Ltd
CD3/4-1BB/PD-L1/ROR1	2021	2021	Tetra-specific Antibody	GNC-035	Sichuan Baili Pharmaceutical Co, Ltd
EGFRvIII/PD-L1/CD3/4-1BB	2021	2021	Tetra-specific Antibody	GNC-039	Sichuan Baili Pharmaceutical Co, Ltd

Abbreviations: BI, Boehringer Ingelheim; BTLA, B- and T-lymphocyte attenuator; CAR-T, chimeric antigen receptor T-cell; CDH17, cadherin 17; EGFR, epidermal growth factor receptor; FRα, folate receptor alpha; FAP, fibroblast activation protein; IRE-1α, inositol-requiring enzyme-1α; PD-L1, programmed cell death ligand 1; ROR2, receptor tyrosine kinase-like orphan receptor 2; TRPV6, transient receptor potential cation channel subfamily V member 6.

In addition, 1359 phase 1 trials of anticancer drugs were initiated, with an AAGR of 23%. Sixty-three phase 1 trials were global multicenter trials, accounting for less than 5% of the total number. Most global multicenter trials were sponsored by Chinese pharmaceutical enterprises (32 of 48 sponsors [67%]). Haihe Biopharma sponsored the most global trials (5 [8%]), followed by BeiGene (4 [6%]), and Novartis (3 [5%]). Institutes that coparticipated with Chinese sites in global multicenter trials were mainly from the US (52 [83%]), followed by Australia (19 [30%]), Taiwan (14 [22%]), and the Republic of Korea (13 [21%]).

Furthermore, an increase of phase 1 trials with a seamless design occurred from 67 in year 1 (2017) to 123 in year 4 (2022) (AAGR, 22%). A total of 358 trials (26%) were biomarker-guided studies with an AAGR of 21%. A master-protocol design was also introduced into those biomarker-guided trials, including umbrella (2 [1%]) and basket (13 [4%]) trials.

## Discussion

This cohort study examined how the reformed and increasingly supportive drug registration regulation in China was associated with the acceleration of the conduct and increase in the number of early-phase trials. The focus of these early-phase trials is transitioning into testing more innovative drugs targeting novel targets, more trials with adaptive designs, and multicenter global trials.

The reformed drug regulation has not only boosted the early-stage development of innovative drugs in China, but was also associated with the pharmaceutical industry value chain supporting a shift from development of me-too to first-in-class drugs in China.^3,4,5,6^ Also supporting the drug development in China is the population of 1.4 billion individuals and the associated many patients and potential trial participants, which was followed by accelerated enrollment of patients into trials.

A study limitation is that the success rates of those phase 1 trials were not calculated because of the relatively narrow study time span, and it deserves to be further investigated in future studies. Nevertheless, obvious improvements in regulatory policies have already had a positive association with innovative drug development, and additional improvements are already being planned. China’s fourteenth 5-year plan (2021-2025) includes increased investment into basic research that aims to further transform drug development from me-too into innovative first in class. All of the previously described points suggest a bright future of innovative drug research and development in China.

## Notes

Supplement. eMethods.
