# Supplementary material for: Evolution of Early-Phase Anticancer Drug Investigations in China
Source: JAMA Oncol. 2022 Sep 8;8(11):1692–4. doi: 10.1001/jamaoncol.2022.3856 (PMC9459900; doi:10.1001/jamaoncol.2022.3856)
Supplement: Supplement. — eMethods. [file jamaoncol-e223856-s001.pdf]

Supplemental Online Content

Wang S, Yu Y, Jiang Y, et al. Evolution of early-phase anticancer drug investigations in China. *JAMA Oncol*. Published online September 8, 2022.
doi:10.1001/jamaoncol.2022.3856

eMethods.

This supplemental material has been provided by the authors to give readers additional information about their work.

© 2022 Wang S et al. *JAMA Oncology.*

eMethods

The following keywords were used for searches on INFORMA database: [(Actual Start Date is from 2017/11/01 to 2021/10/31)] AND [(Trial Phase is I) or (Trial Phase is I/II)] AND (Trial Country is China) AND (Therapeutic Class is Anticancer Products) AND (Disease is Oncology: Solid Tumor)]. Trials were excluded from final analysis if they were translational studies, trials investigating nonpharmacological therapy or adjuvant drugs, or bioequivalence trials on healthy volunteers.

© 2022 Wang S et al. *JAMA Oncology.*
